# The Role of Homocysteine in Pediatric MASLD: A Bipotential Biomarker of Cardiovascular Risk and Liver Fibrosis

**DOI:** 10.3390/life16020191

**Published:** 2026-01-23

**Authors:** Antonella Mosca, Nadia Panera, Giulia Andolina, Luca Della Volpe, Anna Pastore, Maria Rita Braghini, Lidia Monti, Paola Francalanci, Giovanna Soglia, Andrea Pietrobattista, Anna Alisi

**Affiliations:** 1 Hepatology and Liver Transplant Unit, Bambino Gesù Children’s Hospital, IRCCS, 00165 Rome, Italy; luca.dellavolpe@opbg.net (L.D.V.); andrea.pietrobattista@opbg.net (A.P.); 2 Research Unit of Genetics of Complex Phenotypes, Bambino Gesù Children’s Hospital, IRCCS, 00165 Rome, Italy; nadia.panera@opbg.net (N.P.); giulia.andolina@opbg.net (G.A.); anna.pastore@opbg.net (A.P.); mariarita.braghini@opbg.net (M.R.B.); anna.alisi@opbg.net (A.A.); 3 Diagnostic and Interventional Radiology Unit, Bambino Gesù Children’s Hospital, IRCCS, 00165 Rome, Italy; lidia.monti@opbg.net (L.M.); giovanni.soglia@opbg.net (G.S.); 4 Molecular Pathology Research Unit, Bambino Gesù Children’s Hospital, IRCCS, 00165 Rome, Italy; paola.francalanci@opbg.net

**Keywords:** MASLD, homocysteine, non-invasive biomarkers, fibrosis

## Abstract

The increasing prevalence of metabolic dysfunction-associated fatty liver disease (MASLD) in children requires robust, non-invasive biomarkers to enable accurate disease staging and risk stratification. Elevated serum levels of homocysteine (Hcy) have emerged as potential risk factors for cardiometabolic disease in adults, including MASLD. In this observational retrospective study, we investigated the role of serum Hcy levels as a potential biomarker for disease severity and liver fibrosis in a pediatric cohort of 182 children with MASLD. In 89 patients, liver biopsy allowed the classification into metabolic dysfunction-associated steatohepatitis (MASH). Associations between Hcy, metabolic parameters, fibrosis scores, and histological features were examined, and the diagnostic performance of Hcy for liver fibrosis was evaluated using ROC analysis. Multivariate analyses identified elevated Hcy levels as independently associated with HOMA-IR (β = 0.55; *p* = 0.049), TG/HDL ratio (β = 3.23; *p* = 0.002), and liver fibrosis (β = 2.59; *p* = 0.04). Hcy showed a predictive accuracy of 81% for fibrosis. However, the combined diagnostic models of Hcy with non-invasive fibrotic scores (i.e., APRI and FIB-4) or TG/HDL ratio showed only a modest accuracy (AUC = 0.62–0.69). In conclusion, our data suggest that Hcy is associated with fibrosis and cardiometabolic risk. However, these results should be interpreted as exploratory and do not establish homocysteine as a diagnostic biomarker.

## 1. Introduction

Metabolic dysfunction-associated fatty liver disease (MASLD), previously termed non-alcoholic fatty liver disease (NAFLD), is the most common chronic liver disease in children and adolescents worldwide. With a global epidemic of obesity and type 2 diabetes, pediatric MASLD is on the rise, thus posing a future critical issue for public health [1]. Even though MASLD is often considered a benign condition, its progression to metabolic dysfunction-associated steatohepatitis (MASH) involves inflammation and cell damage that can evolve into fibrosis, cirrhosis, and, in rare cases, hepatocellular carcinoma, even at a young age. Early identification of the factors driving this progression is crucial for the timely implementation of therapies and preventing severe long-term outcomes. It is known that children affected by MASLD have an increased risk of developing cardiovascular disease (CVD), the leading cause of long-term morbidity and mortality. Despite the importance of the issue, early identification of children and adolescents with MASLD who are at higher risk of developing CVD is a clinical challenge [2]. Conventional diagnostic methods for CV risk, such as the standard lipid profile, may not be sufficient to detect early signs of vascular dysfunction in this population. In this regard, the triglyceride (TG)/high-density lipoprotein (HDL) ratio has been suggested as a simple and inexpensive biomarker that reflects insulin resistance and atherogenic dyslipidemia [3]. Several studies have demonstrated its usefulness in predicting CV risk in adults; however, its specific role in the pediatric population affected by MASLD remains to be investigated [4,5]. In addition, serum homocysteine (Hcy) levels also appear to be an additional reliable biomarker for assessing CVD risk in specific settings [6]. Indeed, Hcy is a sulfur amino acid whose elevated levels have long been associated with endothelial dysfunction, oxidative stress, and inflammation, all pathogenetic mechanisms that underlie liver disease progression and CVD risk [7]. Hcy has been implicated in the pathogenesis of atherosclerotic complications in patients with metabolic syndrome, a condition that underlies the strong interplay between MASLD and CVD [7]. However, even though hyperhomocysteinemia has been associated with MASLD and insulin resistance in adults, data on its role in pediatric MASLD and its association with cardiometabolic risk in this population are limited [8].

Our study aimed to evaluate the association between the TG/HDL ratio, serum Hcy, and CV parameters in children and adolescents with MASLD and to assess the predictive capacity of these biomarkers for the progression toward liver fibrosis.

## 2. Materials and Methods

### 2.1. Study Population

We enrolled, in our retrospective cohort study, 182 consecutive Caucasian children (mean age 10.89 years; 50% males), with an ultrasound diagnosis of steatosis and persistently elevated serum aminotransferase levels (≥6 months), who were referred to the Liver Unit of the Bambino Gesù Children’s Hospital (Rome, Italy) between January 2015 and March 2025. Following the novel nomenclature and consensus statements [9], all these patients were categorized as MASLD because they had steatotic liver disease (SLD) and at least one of the cardiometabolic criteria without overlapping SLDs (e.g., Wilson’s disease, autoimmune hepatitis, α-1-antitrypsin deficiency, celiac disease, thyropathies, viral, genetic, and metabolic hepatitis, renal impairment, B group vitamin supplements, alcohol and drug use). Of these patients, 89 with moderate/severe steatosis underwent liver biopsy for histopathological evaluation in accordance with ESPGHAN guidelines [10].

### 2.2. Anthropometrical and Biochemical Parameters

Body mass index (BMI) was calculated as weight in kilograms divided by height in meters squared (kg/m^2^). Waist circumference was measured to the nearest 0.1 cm using a non-elastic tape positioned midway between the lower costal margin and the iliac crest at the end of a normal expiration. Blood pressure (BP) was recorded in the right arm using a standard sphygmomanometer; the reported systolic (SBP) and diastolic (DBP) values represent the average of three consecutive measurements. Elevated blood pressure was defined as values exceeding the 95th percentile for age, height, and sex.

Venous blood samples were collected after an overnight fast (at least 8 h). Standard laboratory procedures at the Central Laboratory of the “Bambino Gesù” Children’s Hospital were used to assess the levels of aspartate aminotransferase (AST), alanine aminotransferase (ALT), gamma-glutamyltransferase (GGT), TGs, total cholesterol, HDL, low-density lipoprotein (LDL), fasting blood glucose and insulin, and platelet counts (PLT, expressed in 10^9^/L). Hcy levels were determined via high-performance liquid chromatography (HPLC) with fluorometric detection, as previously described. Our laboratory level is normal, with values <10 µmol/L [8].

### 2.3. Biochemical Scores

The homeostatic model assessment for insulin resistance (HOMA-IR) was calculated as [glucose (mg/dL) × insulin (IU/mL)]/405, and a value greater than 2.5 was considered an index of insulin resistance [11].

The TG/HDL ratio was calculated as TGs (mg/dL) ÷ HDL cholesterol (mg/dL). Values between 2 and 3 indicate a potential metabolic imbalance, while values >3 indicate an elevated risk of CVD. This ratio is beneficial for risk assessment in patients with MASLD, as it directly reflects the atherogenic dyslipidemia typical of this condition [12].

### 2.4. Liver Ultrasound

Fatty liver disease was diagnosed via abdominal ultrasound performed by two experienced radiologists, blinded to the patients’ clinical data, using an Acuson Sequoia C512 ultrasound machine equipped with a 15L8 transducer (Universal Diagnostic Solutions, Oceanside, CA, USA). The “bright liver” pattern was evaluated in comparison to the right kidney parenchyma. Steatosis severity was graded as 0 (mild), 1 (moderate), or 2 (severe).

### 2.5. Liver Biopsy

Eighty-nine children underwent ultrasound-guided liver biopsy under general anesthesia using an 18-gauge automatic needle, following the recent Delphi consensus algorithm [9]. Histological features of MASH, including steatosis, lobular inflammation, ballooning hepatocytes, and fibrosis, were evaluated by a single experienced liver pathologist.

Additionally, each biopsy was independently reviewed by two expert liver pathologists to determine the NAFLD Activity Score (NAS) and fibrosis stage according to the NASH-CRN criteria. Fibrosis was staged on a five-point scale: 0: No fibrosis; 1: Perisinusoidal or periportal fibrosis; 2: Both perisinusoidal and periportal fibrosis; 3: Bridging fibrosis; 4: Cirrhosis [13]. MASH was specifically defined as a NAS ≥ 4 (with at least one point in each category: steatosis, lobular inflammation, and ballooning) and a fibrosis stage ≥ 1.

### 2.6. Assessment of Non-Invasive Fibrosis Scores

Two non-invasive fibrosis scores were evaluated, including the AST/Platelet Ratio Index (APRI) and the Fibrosis-4 Index for Liver Fibrosis (FIB-4) [14]. In particular, APRI was calculated as [AST(U/L)/AST Upper limit of normal(U/L)] × 100/platelet count (10^9^) and FIB-4 as [Age (years) × AST (U/L)/platelets (10^9^/L) × √ALT (U/L)]. All levels of AST were performed at the Central Laboratory of our Children’s Hospital, and the upper limit of normal (ULN) for AST was set at 40 U/L, in accordance with the reference ranges routinely adopted by our laboratory for pediatric patients.

### 2.7. Statistical Analysis

Data are presented as means ± standard deviation, medians, and interquartile ranges (IQR) or frequencies, as appropriate. The continuous variables were analyzed using the ANOVA test (for normally distributed data) and the Mann–Whitney U test (when non-normally distributed). Correlations between metabolic, histological, and one-carbon metabolism parameters were assessed using Pearson’s or Spearman’s coefficients. Multivariate regression analysis was used to test the independent associations of TG/HDL ratio and Hcy with metabolic parameters, as well as histological inflammation, fibrosis, and steatosis, after adjusting for potential confounders (i.e., age and sex). Covariates included in all regression models were selected as potential confounders based on their significance in univariate regression analyses or their biological plausibility.

The diagnostic accuracy of Hcy, TG/HDL ratio, APRI, and FIB-4 was calculated using receiver operating characteristic (ROC) curves. The area under the ROC (AUC) was compared to predict liver fibrosis in MASH patients. The combined AUCs between Hcy and the markers and the scoring system, and the distribution of the values in Log10, were evaluated. Diagnostic performance was assessed by sensitivity, specificity, and positive and negative predictive value (PPV and NPV). A *p*-value of 0.05 was considered statistically significant. Given the limited sample size of the biopsied subgroup, multivariate analyses were considered exploratory and were restricted to a limited number of covariates to minimize the risk of overfitting. No internal validation procedures were performed due to sample size constraints. Analysis was performed using GraphPad Prism 5.01 and MedCalc version 19.1.

## 3. Results

### 3.1. Assessment of Pathological Characteristics of Patients

A total of 182 children diagnosed with MASLD (age range 8.12–14 years) were enrolled in a retrospective cohort study. Among them, 93 (51.1%) patients had mild SLD, while 89 (48.9%) had moderate-or-severe SLD as assessed by ultrasound. According to the established diagnostic criteria, these 89 patients with moderate-to-severe SLD underwent liver biopsy. Histological evaluation showed that, in this group, MASH features were present in 66 patients (Supplementary Table S1), while 23 patients had no evidence of MASH. These data allowed us to classify patients into three distinct groups: (i) the Mild SLD group (N = 93); (ii) the All-SLD group (N = 116), which collectively includes patients with mild (N = 93) and moderate-to-severe SLD (N = 23); and (iii) the MASH group (N = 66). In these groups, we evaluated anthropometric and biochemical parameters, as well as the non-invasive fibrosis scores APRI and FIB-4.

As shown in Table 1, there are no statistically significant differences between Mild SLD and All-SLD patients. Conversely, the MASH group showed statistically significant differences in various parameters compared to the SLD groups. Specifically, MASH patients had higher ALT, AST, insulin, and HOMA-IR values, and lower HDL levels than the mild and All-SLD groups. However, in the MASH group, TG and total cholesterol concentrations were significantly higher, and platelet counts were comparatively lower than those observed in the Mild SLD group.

**Table 1:** Demographic, clinical, and laboratory data for SLD patients, according to disease severity.

Parameters	Mild SLD N = 93	All-SLD N = 116 (Mild, Moderate, Severe)	MASH N = 66	*p* Mild SLD vs. All-SLD	*p* Mild SLD vs. MASH	MASH vs. All-SLD
Sex (M/F)	54/37	67/49	42/24	-	-	-
Age (years)	11 [9.0–14.0]	11 [9.0–14.0]	10.85 [8.12–12.67]	0.40	0.34	0.59
BMI (kg/m^2^)	25.40 [21.28–30.61]	25.70 [21.31–30.70]	26.11 [22.13–28.84]	0.07	0.20	0.27
AST (IU/L)	27.00 [17.25–55.0]	28.00 [21.0–39.0]	45.00 [28.0–62.0]	0.38	**0.001**	**0.001**
ALT (IU/L)	30.00 [17.25–55.0]	33.00 [20.0–60.0]	64.00 [32.75–86.0]	0.44	**0.001**	**0.001**
GGT (IU/L)	16.00 [12.0–27.0]	16.00 [12.0–28.0]	18.51 [14.25–25.0]	0.87	0.94	0.88
Fasting glucose (mg/dL)	87.00 [82.0–92.0]	87.10 [81.0–92.5]	86.50 [78.75–88.0]	0.28	0.15	0.15
Fasting insulin (IU/mL)	17.00 [11.77–25.25]	19.70 [12.0–26.65]	26.41 [18.55–33.85]	0.72	**0.01**	**0.03**
HOMA-IR	3.60 [2.55–5.11]	3.44 [2.42–5.04]	4.12 [2.99–5.61]	0.40	**0.038**	**0.049**
TGs (mg/dL)	81.00 [59.0–115.75]	92.00 [58.0–122.0]	105.00 [69.75–147.0]	0.78	**0.049**	0.09
Cholesterol (mg/dL)	150.000 [136.26–168.50]	151.00 [131.0–163.0]	167 [132.0–198.0]	0.09	**0.03**	0.07
HDL (mg/dL)	42.00 [38.0–51.0]	41.00 [38.75–50.0]	34 [25.0–41.0]	0.10	**0.02**	**0.001**
LDL (mg/dL)	92.00 [78.0–104.0]	92.50 [79.0–104.0]	101 [78.0–113.0]	0.24	0.25	0.09
Platelets, 10^3^/uL	299.00 [220.0–380.0]	279.50 [220.0–399.75]	253 [251.0–320.0]	0.16	**0.045**	0.057
APRI	0.34 [0.25–0.51]	0.35 [0.26–0.57]	0.57 [0.22–0.65]	0.95	**0.032**	**0.041**
FIB-4	1.20 [0.24–1.55]	1.26 [0.35–1.36]	1.35 [0.37–1.82]	0.65	0.07	0.09
SBP (mmHg)	115.00 [103.0–120.0]	115.00 [103.0–120.0]	112.00 [104.0–116.0]	0.23	0.42	0.57
DBP (mmHg)	64.00 [56.25–69.75]	65.00 [57.0–69.0]	69.00 [64.0–77.0]	0.97	0.07	0.051
TG/HDL ratio	1.72 [1.17–2.85]	1.79 [1.22–2.84]	2.37 [1.73–3.95]	0.30	**0.04**	**0.037**
Hcy (µmol/L)	10.00 [7.0–15.0]	10.17 [7.0–17.0]	15.5 [10.21–25.10]	0.09	**0.002**	**0.03**

SLD, steatotic liver disease; MASH, metabolic dysfunction-associated steatohepatitis; F, female; M, male; BMI, body mass index; ALT, alanine aminotransferase; AST, aspartate aminotransferase; GGT, gamma-glutamyl transferase; HOMA-IR, homeostasis model assessment of insulin resistance; TGs, triglycerides; HDL, high-density lipoprotein; LDL, low-density lipoprotein; APRI, AST/Platelet Ratio Index; FIB-4, Fibrosis-4 Index for Liver Fibrosis; SBP, systolic blood pressure; DBP, diastolic blood pressure; Hcy, homocysteine. Data are expressed as an absolute number or median (interquartile P25–P75 range). Statistical significance of differences between groups was analyzed by unpaired *t*-test or Mann–Whitney U test. Bold values indicate statistical significance at the *p* < 0.05 level.

Furthermore, patients with MASH had significantly higher APRI values than those in the SLD groups, whereas no significant differences were observed in FIB-4 indices.

Next, we evaluated CV parameters, including SBP, DBP, TG/HDL ratio, and Hcy levels. As reported in Table 1, there were no statistically significant differences in SBP or DBP values among the groups. It is also noteworthy that a MASH diagnosis was associated with a higher TG/HDL ratio and higher Hcy levels than in the Mild and All-SLD groups.

### 3.2. Correlations Between Hcy and Anthropometric and Metabolic Parameters, Non-Invasive Fibrosis Scores, and Histological Grading in Children with MASLD

The correlation between the mean serum Hcy level and various variables is shown in Table 2.

**Table 2:** Correlation analysis between Hcy levels and cardiometabolic parameters, fibrosis score, and liver histologic features in the MASH subgroup.

	Hcy (µmol/L) Correlation	
Variables	r	*p*
Age (years)	0.06	0.545
BMI (kg/m^2^)	0.22	**0.038**
Fasting glucose (mg/dL)	−0.22	0.288
Fasting insulin (IU/mL)	0.62	**0.008**
HOMA-IR	0.28	**0.006**
TGs (mg/dL)	0.12	0.378
Cholesterol (mg/dL)	0.10	0.547
HDL (mg/dL)	−0.34	**0.021**
LDL (mg/dL)	0.45	**0.011**
AST(IU/L)	−0.11	0.323
ALT (IU/L)	0.068	0.524
GGT (IU/L)	0.094	0.655
Platelets (10^3^)/uL	−0.10	0.942
APRI	0.23	**0.033**
FIB-4	0.063	0.765
SBP (mmHg)	−0.14	0.182
DBP (mmHg)	−0.02	0.848
TG/HDL ratio	0.53	**0.006**
Steatosis	0.09	0.854
Lobular Inflammation	0.24	**0.017**
Ballooning	−0.02	0.844
Fibrosis	0.28	**0.022**
NAS	0.19	0.091

Hcy, homocysteine; BMI, Body Mass Index; HOMA-IR, Homeostatic Model Assessment for Insulin Resistance; TGs, triglycerides; HDL, High-Density Lipoprotein; LDL, Low-Density Lipoprotein; AST, Aspartate Aminotransferase; ALT, Alanine Aminotransferase; GGT, Gamma-Glutamyl Transferase; APRI, AST/Platelet Ratio Index; FIB-4, Fibrosis-4 Index for Liver Fibrosis; SBP, systolic blood pressure; DBP, diastolic blood pressure; NAS, NAFLD Activity score. Correlation analysis using the Spearman Rho method. Bold values indicate statistical significance at the *p* < 0.05 level.

The relationship between serum Hcy concentrations and parameters of cardiometabolic dysfunction, as well as non-invasive scores of liver fibrosis, was first evaluated in all patients with MASLD. As shown in Table S2**,** Hcy levels were positively correlated with fasting insulin (r = 0.28, *p* = 0.04) and HOMA-IR (r = 0.31, *p* = 0.02) and negatively correlated with HDL cholesterol (r = −0.38, *p* = 0.02). Furthermore, circulating Hcy levels showed positive associations with APRI (r = 0.28, *p* = 0.03) and the TG/HDL ratio (r = 0.43, *p* = 0.02).

Subsequently, to assess whether elevated Hcy concentration was associated with histopathological features of the advanced disease phenotype, we conducted Spearman correlation analyses in the subgroup of patients with MASH. In this patient’s subgroup (Table 2), serum Hcy levels correlated positively with BMI (r = 0.22, *p* = 0.038), fasting insulin (r = 0.62, *p* = 0.008), HOMA-IR (r = 0.28, *p* = 0.006) and LDL (r = 0.45, *p* = 0.0011) and negatively with HDL (r = −0.34, *p* = 0.021). Notably, increased levels of Hcy were significantly associated with the fibrosis index APRI and the TG/HDL ratio (r = 0.23, *p* = 0.033; r = 0.53, *p* = 0.006), as well as with histological evidence of hepatic lobular inflammation and liver fibrosis (r = 0.24, *p* = 0.017; r = 0.28, *p* = 0.022). Multivariate regression analysis was performed to further clarify the relationship between these parameters and serum Hcy levels. As reported in Table 3, after adjusting for age and sex, the model revealed that elevated Hcy levels were independently associated with HOMA-IR (β = 0.55; SE = 0.31; *p* = 0.049), with elevated TG/HDL ratio (β = 3.23; SE = 0.94; *p* = 0.002) and histological evidence of liver fibrosis (β = 2.59; SE = 1.24; *p* = 0.04).

**Table 3:** Multivariate regression analysis model between Hcy levels and cardiometabolic parameters, fibrosis score, and liver histologic features in the MASH subgroup.

Variables	β	IF	*p*
Fasting insulin (IU/mL)	0.28	0.43	0.54
HOMA-IR	0.55	0.31	0.049
HDL-cholesterol (mg/dL)	−0.16	0.21	0.07
LDL-cholesterol (mg/dL)	0.25	4.45	0.95
APRI	−12.71	6.92	0.060
TG/HDL ratio	3.23	0.94	**0.002**
Lobular Inflammation	−1.28	1.36	0.35
Fibrosis	2.59	1.24	**0.04**

HOMA-IR, Homeostatic Model Assessment for Insulin Resistance; TGs, triglycerides; HDL, High-Density Lipoprotein; APRI, AST/Platelet Ratio Index. Bold values indicate statistical significance at the *p* < 0.05 level.

In multivariate regression analysis, serum homocysteine levels showed a marginal independent association with liver fibrosis after adjustment for age and sex (*p* = 0.04). Given the limited number of fibrosis events (N = 55; Table S1) in the MASH subgroup.

### 3.3. Evaluation of the Fitness of Hcy Levels in Predicting Liver Fibrosis

**Figure 1:**
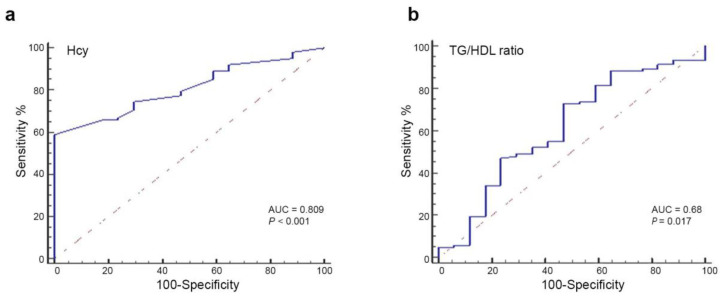
Diagnostic performance of Hcy for predicting liver fibrosis in the MASH patient subgroup. ROC curves of Hcy (**a**) and TG/HDL ratio (**b**), with AUC values and the corresponding *p*-values. The dashed line represents a non-discriminatory test.

Next, we assessed the diagnostic accuracy of Hcy for predicting liver fibrosis specifically in the MASH patient subgroup (n = 66). As shown in Figure 1a, the AUC for Hcy in identifying patients with fibrosis was 0.809 (95% CI, 0.72–0.87; *p* = 0.0001). At the optimal cut-off of >8 µmol/L, Hcy demonstrated a sensitivity of 74.5% [95% CI, 65–83%] and a specificity of 70.6% [95% CI, 44–89%], with a remarkably high Positive Predictive Value (PPV) of 93.8% and a Positive Likelihood Ratio (+LR) of 2.53 (Table S3). Lowering the threshold to >7 µmol/L increased sensitivity (86.3%) but significantly reduced specificity (47.1%). Conversely, a cut-off of >10 µmol/L yielded a specificity of 100% but a lower sensitivity (58.8%).

Conversely, the ROC analysis of TG/HDL ratio (Figure 1b) to distinguish liver fibrosis showed an AUC of 0.68 (95% CI, 0.54–0.71; *p* = 0.017). Using values greater than 1.35, this parameter exhibited a sensitivity of 73.5% (95% CI, 63.8–81.8) and a specificity of 56.25% (95% CI, 30.0–80.2), indicating a poor/moderate discriminatory ability and a predictive performance lower than that of serum Hcy levels (Figure 1b).

**Figure 2:**
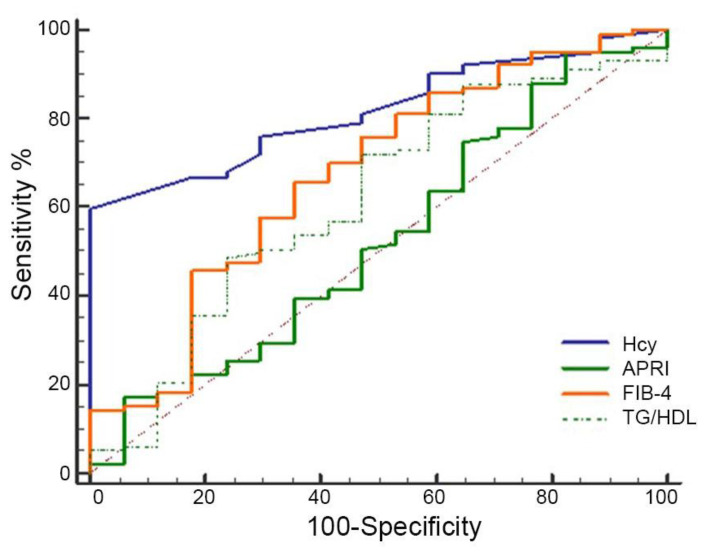
Diagnostic performance of Hcy and non-invasive fibrosis and CV index for predicting liver fibrosis in the MASH patient subgroup. ROC curves of Hcy, APRI, FIB-4, and TG/HDL ratio. The dashed line represents a non-discriminatory test.

Next, we compared the predictive performance of Hcy values with the commonly used serum fibrosis scores and the TG/HDL ratio for predicting advanced fibrosis. In our subgroup of children with MASH, the ROC curves (Figure 2) revealed that the AUC value of Hcy for predicting fibrosis was 0.82 (95% CI: 0.73–0.88; *p* = 0.0001). Conversely, the AUC values were 0.50 (95% CI: 0.41–0.59; *p* = 0.005) for APRI, 0.67 (95% CI: 0.57–0.75; *p* = 0.114) for FIB-4, and 0.63 (95% CI: 0.53–0.72; *p* = 0.034) for the TG/HDL ratio. Among the evaluated indicators, Hcy had the highest AUC, indicating its greatest ability to discriminate between patients with and without liver fibrosis. Compared to Hcy, the predictive accuracy of APRI and the TG/HDL ratio was limited, and the FIB-4 did not reach statistical significance.

**Figure 3:**
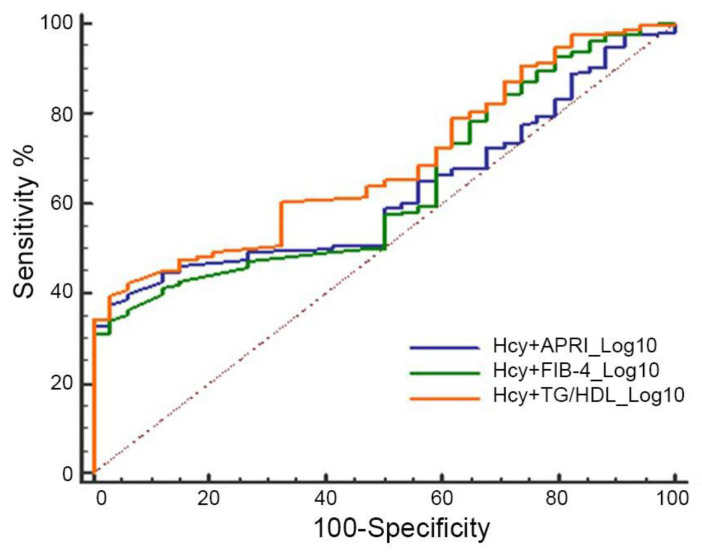
Diagnostic performance of Hcy combined with fibrosis and CV indexes for predicting liver fibrosis in the MASH patient subgroup. ROC curves of Hcy + APRI, Hcy + FIB-4, and Hcy + TG/HDL ratio. The grey dashed line represents a non-discriminatory test.

Finally, we evaluated the AUC of Hcy in combination with each of the other parameters, including APRI, FIB-4, and TG/HDL ratio. Data reported in Figure 3 showed an AUC of 0.62 (95% CI, 0.55–0.68) for Hcy + APRI, an AUC of 0.63 (95% CI, 0.57–0.99) for Hcy + FIB-4, and an AUC of 0.69 (95% CI, 0.62–0.74) for Hcy + TG/HDL ratio, suggesting that combinations of Hcy with other non-invasive indexes for fibrosis and CV risk performed less well than Hcy alone.

## 4. Discussion

The increasing prevalence of MASLD in pediatric populations underscores the need for reliable non-invasive biomarkers to enable accurate disease staging, risk stratification for progression to severe forms such as MASH and liver fibrosis, and differentiation of advanced fibrosis from mild stages.

The principal finding of this study is the association between Hcy levels and liver fibrosis in patients with MASH as compared to those with MASLD and SLD. These results are consistent with our previous results in a different pediatric cohort [15]. Furthermore, in the present study, we identified a direct association between Hcy levels and the histological grade of lobular inflammation and fibrosis (grade > 1), suggesting a causal relationship with the progression of liver damage, which requires further mechanistic exploration [16]. Among the potential mechanisms warranting investigation are endoplasmic reticulum stress, hepatocyte apoptosis, and activation of hepatic stellate cells, all of which have previously been linked to hyperhomocysteinemia [17]. Interestingly, Fu et al. [18] demonstrated that even though higher Hcy levels were associated with increased risk of MASLD and higher liver enzyme levels, genetic liability to NAFLD did not influence Hcy. Moreover, we found that a threshold value > 8 μmol/L allows us to identify children with fibrosis, maintaining a moderate sensitivity. However, these findings should be interpreted as exploratory and do not establish homocysteine as a diagnostic biomarker. Direct comparisons with APRI and FIB-4 did not yield reliable results. Indeed, APRI (AUC = 0.50) was ineffective in our cohort, and FIB-4 (AUC = 0.67) did not reach statistical significance. This result is consistent with a previous study, which partially overlapped with the present cohort (~30%), where these non-invasive scores showed a limited performance [19].

According to Dimitrijevic-Sreckovic et al. [20], our data also reveal an independent association between Hcy levels and both TG/HDL ratio and HOMA-IR, suggesting a synergistic role of these markers in the cardiometabolic derangements that occur in MASLD.

It is necessary to recognize some limitations of our work. Firstly, the cross-sectional design precludes establishing a definitive causal link between Hcy and MASLD progression, allowing only the identification of a strong association. We acknowledge that the imbalance between cases and controls may have reduced statistical power, leading to marginal significance. However, the stability of the diagnostic parameters suggests that the observed association is clinically significant. Secondly, the study was conducted in a single hospital center, albeit highly specialized, and the results need to be validated in larger multicenter studies. Furthermore, not all data have been collected on factors known to influence Hcy levels, such as vitamin status (e.g., folate, B6, and B12) or genetic polymorphisms of the MTHFR gene, which could be confounding factors. In clinical practice, the interpretation of Hcy levels must account for potential false-positive and false-negative scenarios. False-positive elevations of Hcy can occur independently of liver fibrosis in cases of vitamin B12 or folate deficiency, as well as in patients with impaired renal function, which reduces Hcy clearance. Conversely, false-negative results may be observed in patients receiving B-vitamin supplementation or in individuals with specific MTHFR genetic polymorphisms that alter metabolic response. However, we highlight that our results should be interpreted with caution as multivariable models were not adjusted for vitamin status or MTHFR [21]. Moreover, our study may not have detected mild steatosis or may have underreported it; however, the use of liver biopsy as the gold standard in a substantial fraction of the cohort provides robustness to our findings. Finally, an additional limitation is the number of liver fibrosis events (N = 55 patients) in the MASH subgroup, which constrains the statistical power and the stability of multivariate regression models. Consequently, all multivariate analyses should be regarded as not confirmatory. Larger, independent cohorts will be required to validate these findings. [22].

Overall, our findings suggest that Hcy levels show an association with cardiometabolic dysfunction and liver fibrosis, even though its role as a biomarker of disease progression remains unproven [23]. Nevertheless, the evaluation of this marker could be incorporated into clinical practice to identify patients who require more intensive monitoring or a more in-depth evaluation, such as a liver biopsy or elastography.

## 5. Conclusions

In conclusion, our data indicate that Hcy is associated with liver fibrosis in children with MASLD. Moreover, the Hcy levels provide further insight into the integration of cardiometabolic multimorbidities. Further prospective longitudinal studies are warranted to clarify the mechanistic connection between Hcy and fibrosis and to explore whether interventions targeting hyperhomocysteinemia, such as vitamin supplementation, can modify the natural history of MASLD in children.

## Data Availability

The data that support the findings of this study are available from the corresponding author upon reasonable request due to ethical reason.

## Supplementary Materials

The following supporting information can be downloaded at https://www.mdpi.com/article/10.3390/life16020191/s1, Table S1: Histological characteristics of the 89 patients with moderate-to-severe SLD; Table S2: Correlation analysis between Hcy and cardiometabolic parameters and fibrosis score in the children with MASLD; Table S3: Reference ranges and sensitivity/specificity for Hcy.

## Abbreviations

The following abbreviations are used in this manuscript:

ALT	Alanine aminotransferase
APRI	AST/Platelet Ratio Index
AST	Aspartate aminotransferase
BMI	Body mass index
CVD	Cardiovascular disease
DBP	Diastolic blood pressure
FIB-4	Fibrosis-4 index
GGT	Gamma-glutamyltransferase
HDL	High-density lipoprotein
IQR	Interquartile range
LDL	Low-density lipoprotein
HOMA-IR	homeostatic model assessment for insulin resistance
MASLD	Metabolic dysfunction-associated fatty liver disease
MASH	Metabolic dysfunction-associated steatohepatitis
NAFLD	Non-alcoholic fatty liver disease
NAS	NAFLD Activity score
SBP	Systolic blood pressure
SLD	Steatotic liver disease
TGs	Triglycerides
