# Supplementary material for: The Role of Homocysteine in Pediatric MASLD: A Bipotential Biomarker of Cardiovascular Risk and Liver Fibrosis
Source: Life (Basel). 2026 Jan 23;16(2):191. doi: 10.3390/life16020191 (PMC12942120; doi:10.3390/life16020191)
Supplement: Supplementary file 1 [file life-16-00191-s001.zip › life-4073790-supplementary.pdf]

The Role of Homocysteine in Pediatric MASLD: A Bipotential Biomarker of Cardiovascular Risk and Liver Fibrosis

Supplementary Data

Table S1. Histological characteristics of the 89 patients with moderate-to-severe SLD.

**Liver histology features**	**nonMASH (N=23)**	**MASH (N=66)**	***P***
**Steatosis**			
**0**	0	0	**0.002**
**1**	60.9%	4 (6.1%)	**0.001**
**2**	39.1%	42 (63.6%)	-
**3**	-	20 (30.3%)	**0.002**
**Lobular inflammation**			
**0**	5 (21.7%)	1 (1.5%)	**0.001**
**1**	18 (78.3%)	38 (57.6%)	**0.001**
**2**	-	27 (40.9%)	-
**Ballooning**			
**0**	13 (56.5%)	15 (22.7%)	**0.027**
**1**	10 (43.5%)	32 (48.5%)	0.778
**2**	-	19 (28.8%)	-
**Fibrosis**			
0	7 (30.4%)	11 (16.7%)	**0.034**
1	15 (65.2%)	35 (53.0%)	0.125
2	1 (4.3%)	19 (28.8%)	**0.001**
3	-	1 (1.5%)	-
NAS			
**0**	-	-	-
**1**	-	-	-
**2**	9 (39.1%)	-	
**3**	14 (60.9%)	-	
**4**	-	32 (48.5%)	
**5**	-	24 (36.4%)	
**6**	-	8 (12.1%)	
7	-	2 (3%)	

MASH metabolic dysfunction-associated steatohepatitis, NAS NAFLD activity score. Data are expressed in absolute numbers and percentages. Statistical significance of differences between groups was analysed by unpaired t-test or Mann–Whitney U test.

Table S2. Correlation analysis between Hcy and cardiometabolic parameters and fibrosis score in the children with MASLD.

	Hcy (μmol/L) correlation	
**Variables**	***r***	***P***
Age (years)	0.06	0.88
BMI (kg/m2)	0.05	0.81
Fasting glucose (mg/dL)	0.10	0.77
Fasting insulin (IU/mL)	0.28	**0.04**
HOMA-IR	0.31	**0.02**
TGs (mg/dL)	0.21	0.06
Cholesterol (mg/dL)	0.10	0.68
HDL (mg/dL)	-0.38	**0.02**
LDL (mg/dL)	0.21	0.06
AST(IU/L)	0.10	0.11
ALT (IU/L)	0.19	0.82
GGT (IU/L)	0.09	0.91
Platelets (10^3)/ uL	0.04	0.97
APRI	0.28	**0.03**
FIB-4	0.19	0.31
SBP (mmHg)	0.17	0.54
DBP (mmHg)	0.15	0.65
TGs/HDL ratio	0.43	**0.02**

MASH metabolic dysfunction-associated steatohepatitis, BMI Body Mass Index, HOMA-IR Homeostatic Model Assessment for Insulin Resistance, TGs triglycerides, HDL High-Density Lipoprotein, LDL Low-Density Lipoprotein, AST Aspartate Aminotransferase, ALT Alanine Aminotransferase, GGT Gamma-Glutamyl Transferase, APRI AST/Platelet Ratio Index, FIB-4 Fibrosis-4 Index for Liver Fibrosis, SBP systolic blood pressure, DBP diastolic blood pressure. Correlation analysis using the Spearman Rho.

Table S3. Reference ranges and sensitivity/specificity for Hcy.

Hcy Cut-off	**Sensitivity (%)**	**95% CI**	**Specificity (%)**	**95% CI**	**PPV (%)**	**NPV (%)**	**+LR**	**-LR**
>7	86.27	78.0 – 92.3	47.06	23.0 – 72.2	90.7	36.4	2.03	0.29
>8 (*)	74.51	64.9 – 82.6	70.59	44.0 – 89.7	93.8	31.6	2.53	0.36
>9.5	66.09	55.6 – 74.8	82.35	56.6 – 96.2	95.7	28.6	4.12	0.42
>10	59.22	48.6 – 68.5	100	80.5 – 100	100.0	28.8	—	0.41

Hcy homocysteine, CI confidential interval, PPV Positive Predictive Values, NPV Negative Predictive Values, +LR Positive Likelihood Ratio, - LR negative likelihood ratio. * values >8 μmol/L represent the optimal clinical balance (Youden's Index) for this pediatric cohort. The inclusion of Positive Predictive Values (PPV) and Negative Predictive Values (NPV) further demonstrates the clinical robustness of our findings, with a PPV of 93.8% for the primary cut-off.

.
